# A Human Monster

**Published:** 1876-01

**Authors:** A. R. Kilpatrick

**Affiliations:** Navasota, Tex.


### A HUMAN MONSTER.

A. R. KILPATRICK, M. D.

"Truth is strange; stranger than fiction."

A long time ago—way back in the forties—what I am going to relate, took place in the State of Mississippi, not very far from Natchez. If I am a little prolix or verbose, I hope the singularity of the occurence will relieve the reader from *ennui* and restlessness.

A gentleman named Bond, who was both lawyer and planter, resided in that beautiful City of the Bluffs, Natchez, and owned a large cotton plantation below, in the county of Wilkinson, under the supervision of good overseers, or managers, as they are sometimes called, by way of distinction over common overseers on small farms. Mr. Bond visited his plantation only a few times each year, as his managers were men of intelligence, fully capable of carrying on the business without the aid or direction of the proprietor; and sometimes those managers possessed more knowledge and skill about the conduct of a farm than the owners.

The overseers had some knowledge of the diseases amongst the negroes, and always were supplied by the proprietors with the staple medicines mostly needed in their treatment, dispensing them to the nurse who carried out their instructions. Generally the nurses were trusty, old women, who had reared large families, and had had the care of many cases under the directions of master and mistress and the family physician in years gone by. Mr. Bond's place was a model of order, regular discipline and neatness; everything was conducted well, in a semi-military style, but no cruelty or tyranny.

Under these circumstances the custom was to engage the services of a physician at a regular stipend, payable at the end of the year, or at so much for each visit, an account of which was kept both by the doctor and the overseer, until the time of settlement. Very often the judgment and preference of the overseer was acquiesced in by the proprietor, and the doctors paid court to the employee instead of the owner. When the estate was very large, the proprietor would employ a physician at a certain salary, to live on the place and devote his services to the treatment of the slaves, granting permission for him to attend other outside calls where they did not interfere with his home business. Very often, these places were sinecures, the services being light and simple, and some needy relative was put in the place, or some young practitioner who was aspiring to a larger sphere of business. The salaries ranged from a few hundreds of dollars a year, to one or two thousand.

Mr. Bond employed Dr. Dungan, an elderly gentleman of rare attainments, who had served many years as surgeon in the U. S. Navy; who had traversed the globe and visited all quarters, in both hemispheres, and had retired to a quiet country practice where the pay was good and the responsibilities not so onerous as in the city. He was genial and sociable and when the stimulus of the occasion roused him, could "many a tale unfold;" tell of many "hair-breadth escapes," and "of moving accidents by flood and field." And, in the families of the rich who occupied the country around, the doctor often found appreciative auditors. The doctor loved fine horses, good cigars, good dinners and fine wines, of which he professed to be a judge, as he had tasted all vintages in their native habitat.

In purchasing medicines for the plantation, Mr. Bond laid in largely of staple articles, and always bought castor oil by the cask, or barrel; the last barrel had been bought from Mr. Fox, the leading druggist of Natchez, and sent down to the plantation on a wagon with other things. When the old supply was exhausted and this barrel was tapped by the overseer, he was surprised to see an entirely different fluid run out. He examined the brand on the barrel-head and found it "Castor Oil, St. Louis, Mo." It so happened that the doctor was there, being called to see some cases, and had ordered oil to be administered to one or two of them. The overseer showed the liquid to him and asked his opinion as to what it was? After smelling, scanning and tasting it, he pronounced it wine, excellent port wine; it had the real flavor, the true bouquet of genuine old Port, of which he had so often drank when in Europe, and on his cruise in the Mediterranean Sea, in the year 18—! Here was richness; he drank and told the overseer to drink, which he did with very little urging; he fell in as easily as Adam did at the first. Many of the neighbors, high and low, took a pull at the Old Port. Not long after this, Mr. Bond came down, and he drank of it; and when he returned home, took a demijohn full with him and regaled sundry of the gentry in and around Natchez with the delicious potation. I would merely remark, in parenthesis, that the doctor very often found his business to call him to the plantation, and every time he was sure to try that Port and smack his lips and discuss its merits. It made him as mellow as Tam O'Shanter, or sonter Johnny, and many a thrilling narrative, or tender legend was wasted in the ear of the drowsy overseer.

But time sped on and with it ebbed the Old Port. Thousands of surmises and speculations had been canvassed as to how the wine came to be so nauseously labeled, and as to where it came from. Finally it was all drank up and in spite of coaxing and tilting the barrel, no more could be got out of it, and they were all sorry.

After a month or two, the cook-woman called on the overseer for a barrel to put soft soap in, to send up by the wagon to Mistress, at Natchez, for the washwoman to use. He went to the store-room and after examining several, finally selected that wine barrel and put it out for her, with instructions that when the hands came in at noon for dinner, to tell Ned to remove the head carefully, and after she had cleaned it and put in the soft soap, for him to replace the barrel-head securely. Ned was a carpenter, had skill in the use of tools, and made gates, repaired wagons and did other jobs of the kind.

The yard was full of negroes; some eating, some talking and laughing, some dozing in the shade; the overseer and the doctor were in the house eating dinner when Ned loosened the hoops and knocked in the barrel-head. He reached down to get the pieces when he saw something which made "his two eyes, like stars, start from their spheres; his knotted and combined locks to part, and each particular hair to stand on end, like quills upon the fretful porcupine!" He sprang back from the barrel as if attacked by a rattlesnake, his eyes and mouth open as wide as he could stretch them, and both hands raised high over his head, holding pieces of the heading, exclaiming: "My God! folks, look y'er! what dis yer?" In an instant, one and all ran, impelled by that eager curiosity characteristic of the race, and looked in. In the dregs and sediment of the wine they saw something, at first indefinable, but by turning it, they found

A MONSTER!

A double-headed and four-legged negro child, in the slime and slush, seeing which they all began to retch and vomit. Some one in breathless haste ran in and called the doctor and overseer out to see what that was they found in the barrel. The motley crowd presented the appearance of a shipload of land lubbers in a rough sea, all casting up their dinners. The doctor looked and gazed in wonder, so did the overseer; and when they thought of having drank the fine old Port down to the lees, each one hunted a resting place for his head and their revolting stomachs discharged their contents.

Ever after that, the easiest way to offend Dr. Dungan was to ask him about that old Port. The overseer was testy about it, and in time absolutely denied ever tasting it. Mr. Bond, also, was very reticent on the subject.

Dr. Dungan had the child taken out, washed clean and put it in a glass jar filled with alcohol, and carried it to Woodville, the county seat, where it was on exhibition several weeks in the drug store of Dr. C. H. Stone, who afterwards sent it to the Professors of the Medical College in New Orleans. The body was opened and the viscera examined. A full report was published in the first or second volume of the *N. O. Med. Journal*, and with it was given a lithograph engraving of the child, or children.

They had reached the full term of gestation; females; black; joined side to side from the shoulders to the hips; two heads, four legs, two arms, one to each outer side of the child, with hands perfect, and a false, imperfect, third arm projecting upward from the seam of junction behind, devoid of hand or fingers. For further particulars I refer you to the published account in the *N. O. Medical Journal*. The monster is now in the Museum of the College, or at least I saw it there two years ago. The heads are covered with full suits of hair or wool, whichever you please to term it.

The druggest, Fox, in Natchez, could never find out how it came there. He had bought the barrel, as a barrel of Castor Oil, and had never tapped it before selling it. Probably some accoucheur had had the monster put in an old oil barrel, filled it with whiskey, and headed it up with the intention of sending it to some Museum, when he may have died suddenly, or been blown up on a steamboat before he accomplished his purpose, and the barrel got misplaced.

It is an o'er true tale, and I write from personal knowledge of the circumstances.

NAVASOTA, TEX.

